# Supplementary material for: Are carbon emissions trading and green financial instruments synergistic? -Comprehensive quantitative research based on content analysis
Source: PLoS One. 2024 Mar 7;19(3):e0298601. doi: 10.1371/journal.pone.0298601 (PMC10919601; doi:10.1371/journal.pone.0298601)
Supplement: S1 Data — (ZIP) [file pone.0298601.s001.zip › Data/地级市Z_F工作报告.zip!/╒π╜¡/└÷╦«╩╨/└┤╫╘╣1⁄2╓┌║┼í╛┬φ┐╦╩2╛▌═°í┐_2015.docx]

各位代表：

　　现在，我代表市人民政府向大会作工作报告，请予审议，并请市政协委员和其他列席人员提出意见。

一、2014年工作回顾

　　过去一年，是丽水全面确立并实施“绿水青山就是金山银山”战略指导思想的开局之年。面对宏观经济发展新常态，全市上下在省委、省政府及市委的坚强领导下，以推进绿色生态发展、建设美丽幸福新丽水为导向，扎实做好各项工作，实现经济社会持续稳步发展。

　　(一)生态文明和经济建设在转型升级中协同推进。

　　坚持在转型升级中求发展，努力实现经济、社会、生态综合效益最大化。全省排名的34项主要经济指标增速，有10项居全省前三。全市生产总值增长7%，达到1051亿元，突破千亿大关。一般公共预算收入增长9.8%，达到80.96亿元，提前一年实现“十二五”规划目标。生态环境约束性指标均实现省定目标，在全省率先创成省级生态市，成功列入国家生态文明先行示范区第一批建设名单，云和、遂昌创成国家级生态县。

　　治水治气、治城治乡统筹联动，倒逼转型升级。打好“五水共治”攻坚战，全民治水，治污先行，整治垃圾河140条710公里、黑臭河60条378公里。市区丽阳坑、好溪堰水系整治一阶段工程基本完成。加快城镇截污纳管，新建污水管网142公里。完成农村生活污水治理889个村。全面完成禁养区养殖场关停搬迁工作。全市地表水Ⅰ—Ⅲ类水比例达到98.96%，跨行政区域交界断面水质达标率100%。缙云获得全省“五水共治”“大禹鼎”。开展大气治理，淘汰黄标车及老旧车12688辆，空气质量优良天数达到310天。深入推进“三改一拆”，累计拆除违法建筑332.58万平方米，完成“三改”623.11万平方米，云和创建“无违建县”通过市级初验。废弃矿井治理率94.7%。深化“六边三化三美”，新增优质林分公益林76.92万亩、重点防护林2.88万亩，新建美丽乡村风景线10条。

　　“四换三名”加快实施。淘汰关停不符合生态环保要求的企业110家，盘活存量土地4238亩、腾出用能总量1.4万吨标准煤。成立机器人产业联盟，实施“机器换人”示范项目77个。农村电子商务“遂昌赶街模式”引起广泛关注并被复制推广，7个县(市、区)入选中国电子商务百佳县，全市实现电子商务零售额76.7亿元，增长53.6%。新增驰名商标、地理标志证明商标、集体商标27件，新增省级名牌和省著名商标36件。

　　创业创新活力进一步激发。改革商事登记制度，促进新增市场主体2.48万户，其中新增企业4410家。推动企业对接多层次资本市场，在“新三板”市场成功挂牌企业5家，在浙江股权交易中心挂牌企业达到132家，在天津渤海商品交易所挂牌上市的大宗商品达到8种。新创省级高新技术特色产业基地2个，新增省级企业研究院和研发中心24家、国家重点支持高新技术企业51家，主导修订国家标准和行业标准5项。

　　有效投资进一步扩大。引进亿元以上大项目119个，其中5亿元以上大项目27个。全市实际利用内资286.5亿元、外资1.78亿美元，浙商回归到位资金105.3亿元，固定资产投资665.08亿元、增长16.6%。浙北至福州特高压输变电、杭丽热电集中供热等项目建成投入运行，金丽温铁路扩能改造路基工程基本完成，50省道莲都段、松阳段等一批高等级公路建成通车，衢宁铁路开工建设。统筹稳出口、促消费等工作，外贸出口增长11.1%，社会消费品零售总额增长13.2%。

　　(二)生态产业在集聚提升中加快发展。

　　生态农业特色优势不断凸显。新建粮食生产功能区6.19万亩、省级现代农业园区21个，新发展家庭农场1372家。在杭成功举办丽水生态精品农博会。创建“丽水山耕”区域公用品牌，新增“三品”认证农产品191个，新增中国生态原产地保护产品4个。茶产业、食用菌综合产值均突破百亿大关。所有县(市、区)均建成农产品安全快速检测室，对公众免费开放。

　　生态工业培育有新推进。以生态工业负面清单制度为核心的产业政策进一步完善，生态产业集聚区、开发区(园区)生态化改造取得阶段性成效，丽水开发区晋升为国家级经济技术开发区，成为省级循环化改造示范试点园区、生态化建设试点园区。结构调整迈出新步伐，战略性新兴产业、高新技术产业和装备制造业增加值增速，分别高出规上工业增加值增速1、2.5和3.3个百分点，新产品产值增长23.2%。推进“两化”深度融合，绿谷信息产业园入驻企业68家。

　　生态服务业协调发展。生态旅游名城建设加快推进，创建国家5A级旅游景区有实质性进展，新增4A景区2家、省级旅游度假区1家，全市实现旅游总收入339.58亿元，增长27.5%。农家乐综合体模式获“2014中国全面小康十大民生决策奖”。遂昌入选“2014‘美丽中国’十佳旅游县”。获全国第一个“气候养生之乡”称号，养生养老行业地方标准2项发布实施、5项通过评审。年度重点实施的11个服务业集聚区建设顺利推进，丽水现代商贸中心、无水港一期、浙西南绿色农产品物流中心等项目投入使用或基本完工，服务业投资增长23.2%。

　　(三)深化改革在有序推进中重点突破。

　　三大全国改革试点深入推进。农村金融改革试点与农村产权制度改革有机结合，建立农村产权交易服务体系，实现农民基本产权可抵押，新增“三权”抵押贷款19.23亿元；实现新型农村金融组织县域全覆盖，在全国率先实现农村基本金融服务不出村。低丘缓坡综合开发利用试点与山海协作产业园建设有机结合，拓展了产业发展空间。推进扶贫改革试验区建设，云和试点经验在全市推广，异地搬迁农民20932人，完成集体资产股份化改造2423个村。

　　一系列体制机制改革稳步推进。推进“一县一主题”社会事业综合改革试点，6个民办教育、医疗项目开工建设。推进旅游全域化综合改革，设立市旅游委员会，组建瓯江生态旅游景区管委会、市旅游投资发展公司。推进投融资体制改革，组建华侨投资管理公司，开展民间融资服务中心试点。推进国有资产管理机制改革，加大市直政府性资产清理和盘活力度，盘活政府性资产收益3.01亿元。推进丽水开发区体制机制改革，强化产业培育功能和社会事务管理职能。推进政府机构改革，整合组建市场监督管理局、卫生和计划生育委员会、食品药品与质量技术检验检测院。

　　(四)城乡面貌在精细化建设中更加秀美。

　　城市品位进一步提升。在全民创卫的努力中，中心城市通过国家卫生城市创建技术评估，并被评为“2014中国十大特色休闲城市”，成为省级历史文化名城。完成花园路综合改造、高速丽水出口富岭连接线整治、紫金路和南明湖沿线亮化、规划馆改造等一批城市亮点项目，完成老小区改造8个、农贸市场改造5个、背街小巷整治317条，城区街头绿地建设项目获中国人居环境范例奖。美丽县城“六个一”项目建设加快推进。深化城市治堵，市区新增停车位5102个、公交站点40个、优化公交线路9条，在全省率先实现公共自行车租赁市域“一卡通”。

　　美丽乡村建设向纵深推进。莲都碧湖镇等9个镇入选新一批全国重点镇，青田洞背村、缙云鼎湖村入选“中国最美村镇”20强。新创市级美丽乡村示范村20个、最美乡村3个，创成新农村电气化市。加强历史文化村落保护利用，松阳杨家堂等56个村列入第三批中国传统村落名录，居全省之首。

　　(五)民计民生在共建共享中持续改善。

　　民生保障不断加强。城乡居民收入进一步提高，全市城镇居民人均可支配收入30413元，增长8.6%；农村居民人均可支配收入13635元，增长12%。全市十方面大事、市区十方面实事基本完成，全市民生类支出165.9亿元，增长14.8%，占一般公共预算支出的76.4%。新增城镇就业2.73万人，城镇登记失业率2.92%。城乡居民医疗保险实现全市统筹，覆盖全市城镇职工和城乡居民的大病保险制度建立实施。新增养老、医疗、工伤、生育、失业保险15.89万人次。新建养老服务机构11家、居家养老服务照料中心550个。新建成保障性安居工程住房3060套。

　　社会事业进一步发展。7个县(市、区)通过全国义务教育基本均衡县评估，高考纯文理第一批上线人数达1545人，创历史新高。医药卫生体制改革有序推进，全面实施基本药物零差率销售，药品累计让利3.8亿元，紧密型乡村卫生一体化管理率达91.2%。通济堰入选首批世界灌溉工程遗产名录，庆元香菇入选中国重要农业文化遗产保护项目，新增景宁畲族婚俗等国家级“非遗”项目3个，龙泉被评为全国文化先进市。新建农村文化礼堂80家。成功举办丽水国际轮滑公开赛等6项国际国内赛事。创新社会管理与建设，开展“百名市县领导包百案”活动，信访总量下降34.2%，安全生产事故起数、死亡人数连续11年下降，食品安全群众满意率连续6年全省第一，群众安全感满意率98.2%。全市党政军民奋力抗击50年一遇“8·20”全流域性洪灾，做到人员“零伤亡”，灾后恢复重建迅速到位。人口计生、老龄、妇女儿童、残疾人、民族宗教、侨台外事、慈善、移民等事业取得新进展，工青妇等群团工作和国防动员、人民防空、双拥优抚等工作取得新成效。

　　(六)政府自身建设在党的群众路线教育实践活动中不断加强。

　　政风政纪建设扎实推进。坚持以上率下，认真查摆和整改“四风”问题。坚持依法行政，坚持重大事项向人大报告和政协通报制度，交由政府系统办理的172件人大代表建议和361件政协提案全部办结。开展政府议定事项“集中督查、狠抓落实”专项行动，清理议定事项825件，截至目前落实率98.6%。常态化开展“电视问政”。推进预算绩效管理，厉行节约，“三公”经费下降37.9%。加强审计监督和行政监察，避免和挽回经济损失4.76亿元。强化专项整治，查处“吃拿卡要”、“慵懒散慢”问题230起448人，减少收费、罚款项目83项，清理清退“吃空饷”125人。

　　政务环境进一步优化。加快“四张清单一张网”建设，政府部门权力事项精简比例达到53.7%。创新审批“大代办”服务，累计代办项目345个，涉及投资388.06亿元。创建市级“中介服务超市”，51家审批中介入驻实现规范管理。政府和企业投资项目审批流程分别由235、145个工作日，缩短到31、22个工作日，实行审批服务超时默认和缺席默认制度，在打造“最佳投资环境之市”上取得新进展。

　　各位代表，一年来的成绩来之不易。这是市委正确领导的结果，是全市人民团结奋斗的结果。在此，我代表市人民政府，向全市人民以及广大建设者，向人大代表、政协委员，各民主党派、工商联、人民团体和社会各界人士，向驻丽部队官兵和省部属驻丽各单位，向关心和支持丽水发展的海内外朋友们，表示衷心的感谢！

　　我们也清醒地看到，政府工作还有不少差距：在转型升级和经济下行的双重压力影响下，地区生产总值、工业增加值等经济指标低于预期目标，新旧发展动力转换“青黄不接”问题比较突出，部分企业生产经营比较困难，培育新的增长点亟需付出更大努力。对照国家生态文明先行示范区建设标准，生态经济发展还不够充分，生态环境质量继续保持全国全省前列压力在加大。对照人民群众对美好生活的新期盼，民生保障迫切需要解决的问题还有很多，特别是城乡居民持续增收基础还不稳固，低收入群众增收难度加大；城市宜居程度有待进一步改善，市区路网和地下管线有待进一步完善，有的区块拆迁安置还没有落实到位。对照转变作风的新要求，“四风”问题在一些部门和地方还有发生，一些政府工作人员改革意识、责任意识、法治意识不强，“为民、务实、清廉”及“三严三实”的长效机制建设需要不断加强。对此，我们将高度重视，切实加以改进。

二、2015年工作的目标和任务

　　2015年是全面深化改革的关键之年、全面推进依法治国的开局之年，也是全面完成“十二五”规划、研究制定“十三五”规划的重要节点。从我市来说，还是新常态下全面推动“绿水青山就是金山银山”战略指导思想进一步“落地”，加快建设美丽幸福新丽水，努力为建设“两富”、“两美”浙江作出新贡献的重要一年。综合研判形势，既要看到新常态下中央及我省更加注重经济增长质量和生态文明建设，通过绿色生态发展，尽快“富”起来、更加“美”起来、摘帽“欠发达”、实现“绿富美”已成为丽水发展的新使命；又要看到面对新常态以及宏观经济“三期叠加”大背景，丽水发展面临具有自身阶段性特点的“三期叠加”(结构调整集中期、生态产业培育期、深化改革攻坚期)以及“高铁时代”所带来的机遇和挑战。必须通过深刻认识新常态，主动适应新常态，积极引领新常态，努力变挑战为机遇。必须保持定力、同心协力、集中精力、精准发力，努力变生态潜力为生态生产力。必须加快推动发展动力从传统增长点转向新的增长点，在不纠结于一时速度高低的同时，发挥创造性，保持进取心。

　　今年政府工作的总体要求是：以省委“八八战略”为总纲，坚持“绿水青山就是金山银山”战略指导思想，把握新常态，抢抓新机遇，坚持以“稳中求进、转型发展”为工作主基调，加快建设生态保护和生态经济“双示范区”，全力抓好生态、经济、平安、民生等“报表”，努力建设美丽幸福新丽水。

　　建议今年全市经济社会发展主要预期目标为：地区生产总值增长7.5%左右；农业增加值增长3.5%；规上工业增加值增长8%；服务业增加值增长7%；固定资产投资增长15%以上；出口总额增长7.5%；社会消费品零售总额增长11%以上；一般公共预算收入增长7.5%。

　　做好今年的工作，必须突出绿色生态发展、转型发展、创新发展，大力推进生态产业强市，加快建设美丽幸福新丽水这条“主线”。必须强化生态空间管治这条“红线”。必须把握好力争发展速度不低于全省平均水平这条“基线”。必须把百姓增收、生态良好、社会平安作为经济社会发展的“三条底线”，尤其要把生态良好作为第一“底线”，确保节能减排相关约束性指标及空气质量优良天数、地表水Ⅰ—Ⅲ类水比例均完成省下达的目标任务；确保城乡居民可持续增收，城镇和农村居民人均可支配收入分别名义增长8%和10%；确保实现平安市创建目标。

　　(一)围绕全面推动“绿水青山就是金山银山”战略指导思想进一步“落地”，强化绿色生态发展的各方面保障。

　　强化绿色生态发展的制度保障。把严格生态红线空间管治作为抓经济、抓发展的基本依据，争取用3年时间，推进工业企业整合重组、整治转型，努力实现生态产业集聚区、开发区(园区)以外基本无工业，园区之内基本没有非生态工业。强化生态执法、生态考核、生态督查“三位一体”的制度体系建设。严格执行新《环保法》，对环境违法行为“零容忍”，逐步推进生态环境审计和终身责任追究制度。加强生态巡查，持续抓好违建厂房拆除及破旧厂区改造、涉及工业排污截污纳管管网建设、污染企业整治、明察暗访等“四个每周一报”工作制度的落实，使之成为生态产业强市的“先手棋”。完善生态约束和生态激励有机结合的制度体系，深化和扩大排污权有偿使用，落实绿色信贷、绿色保险、绿色采购等环境经济政策。

　　强化绿色生态发展的规划保障。抓好“十三五”发展规划及专项规划编制，使之成为践行“绿水青山就是金山银山”的路线图、任务书、项目库。突出“空间一张图”，以加强生态红线空间管治为前提，探索生态、产业、空间、城镇、交通等规划“多规合一”。突出“市域一盘棋”，以组织实施新一轮《丽水市城市总体规划》为契机，构建中心城市与组团布局县域小城市的紧密型关系，推进县域经济向市域经济转型。突出“产业一条龙”，积极对接全省七大万亿产业，以深化龙头企业带动的产业链培育和战略性新兴产业培育为重点，完善生态产业规划体系。

　　强化绿色生态发展的项目保障。深化重大项目、重大工程、重大政策研究和谋划，强化项目支撑，争取我市“三个重大”更多地进入国家和省计划盘子。突出抓好637个总投资2265亿元的生态文明先行示范区“5+1”重点项目库、616个总投资2922亿元的生态产业项目库、120个总投资1000亿元的重大前期项目库。主攻项目落地，实行重点项目推进每月通报排名、“五率”考核和破解难题“部门首办、重点交办、联合督办”机制。强化华东绿色能源基地、绿色能源输送网和绿色生态屏障建设，力争金丽温输气管道、市区天然气利用项目建成通气，确保一批光伏发电项目建成投产，一批风力发电项目开工建设，推进缙云抽水蓄能电站前期。全市抓好重点建设项目260个，年度计划投资220亿元。

　　强化绿色生态发展的人文保障。弘扬生态文化，提升干部群众、市场主体的生态文明素养，对市直政府机关公务员及全市规上企业家进行“全员轮训”。通过抓好绿色生态发展主题培训，深化认识不考核GDP是不唯GDP，不要“黑色”、“灰色”GDP，使精心培育绿色GDP进一步成为共识；深入落实生态红线空间管治和“负面清单”管控，使之成为政府和企业的操作规范、社会的监督规范。

　　(二)围绕培育新的增长点，大力推进生态产业强市。

　　狠抓生态农业基本点。深入实施生态精品农业“361”工程，做好“接二连三”文章，加快建设一批生态精品农业种养区、特色优势农产品加工区、农业休闲发展区和农业生态功能区，培育一批标准化、基地化、产业化的生态精品农业产业带、产业群。加快促进“丽水山耕”成为覆盖全区域、全产业、全品类的生态农业“金标签”，加快构建农业科技服务体系和农产品质量安全追溯体系、监管体系。大力发展“三位一体”农村合作经济。积极培育新型职业农民。积极推进省级生态循环农业示范县创建。支持莲都抓好国家农业综合开发现代农业园区试点项目。新建粮食生产功能区5.9万亩、现代农业园区15个，实现农产品加工园区供地1500亩。新培育生态精品农业示范乡镇15个、示范家庭农场200家、示范企业和示范合作社50家、“三品”认证农产品60个、生态精品农产品200个。

　　狠抓生态工业启动点。坚持传统产业改造提升和培育发展战略性新兴产业两手抓，重点组织实施市级100个以上战略性新兴产业项目库，力争战略性新兴产业增加值增长9%、新产品产值增长12%。聚焦12个省级以上生态产业集聚区、开发区(园区)主战场，力争丽水开发区列入国家循环化改造试点园区，强化促投资、促投产、促开工“三促”推进，低效产业、低效闲置用地、低质招商项目“三低”治理，名企、名品、名家“三名”打造，新产品、新装备、新服务“三新”开发和推广应用，加快实现高排放、高消耗、高投入“老三高”向产业高端、技术高新、投资高效“新三高”转变。抓好生态产业集聚区、开发区(园区)整合提升，推进并完成全市乡镇工业功能区纳入生态产业集聚区、开发区(园区)统一管理开发，实现规划统编、开发统筹、管网统建、污染统治。

　　狠抓生态服务业增长点。大力发展生态旅游、养生养老等绿水青山型支柱产业。把握浙皖闽赣将四省交界地区规划为国家级生态文化旅游示范区的契机，深入实施生态旅游“1311”行动计划，着力打造“第一战略支柱产业”和“生态旅游第一市”。加快国家5A级旅游景区创建，推进千峡湖旅游综合体等重点项目，启动国家“竹海公园”、华东药用植物园、瓯江生态旅游景区建设。大力发展乡村休闲旅游，新建农家乐综合体10家。全市旅游总收入增长25%。加快培育养生养老产业，重点推进“112”养生养老项目建设及一批养生乡村的改造提升。推进文化产业创意化发展，抓好“六个一批”文化项目建设，加快培育剑瓷、石雕两大“百亿文化产业集群”。加快发展现代商贸、物流和金融业，重点抓好13个省市级服务业重点集聚区，加快推进无水港二期、华侨总部大楼、浙西南工贸综合体等重点项目。

　　狠抓生态特色小镇的谋划和建设。以全省加快规划建设一批特色小镇为契机，以企业主体、资源整合、项目组合、产业融合为原则，创新思路、加强规划、狠抓落实，积极谋划和建设一批体现丽水生态优势，产业、文化、旅游功能叠加的特色小镇，以新理念、新机制、新载体推进产业集聚、产业创新和产业升级。力争全市谋划特色小镇30个以上。

　　狠抓生态经济与信息经济的全面融合发展。紧紧抓住信息经济崛起的时间窗口，研究制定发展政策，制定“互联网+”行动计划，推动“3+3”生态产业体系的每一个产业都要用好互联网思维。打造“两化”深度融合示范区域、示范项目和示范企业，支持丽水开发区加快推进省“两化”融合绿色安全制造信息化试点，抓好绿谷信息产业园、海创园、机器人产业基地建设。加快丽水电子商务产业集聚园建设。加强与阿里巴巴集团战略合作，办好“网上生态精品农博会”、“淘宝村”高峰论坛，建成1000个村级赶街网点和9个县级农村电子商务营运中心，发展更多的“淘宝村”。促进跨境电子商务发展。支持发展大数据和云服务产业，探索开展智慧旅游、“健康云”养生养老等多样化“智慧应用”。积极推进信息惠民国家示范城市建设。加强3D打印等新技术的应用研究。

　　(三)围绕在转型中成型、在提质中扩量，坚定不移打好转型升级“组合拳”。

　　以“五水共治”、“三改一拆”和联动推进城乡环境综合整治，持续倒逼转型升级。大力推进全省统一部署的“消灭黑臭河、劣V类水三年行动”、“燃煤电厂清洁排放技术改造三年行动”、“农业‘两区’土壤污染防治三年行动”、“创无违建县三年行动”。完善河长督办问责制度，健全河岸一体化保洁机制，巩固“清三河”成果，实现河清水洁可游泳、岸绿景美可游览。严格执行空间、总量、项目“三位一体”环境准入制度，综合运用法律、经济、行政、舆论、综合监督等多种手段，以“快刀利剑”依法严厉打击企业违法搭建、偷排、直排行为，通过淘汰一批、拆改一批、关闭一批、整治一批，使环境突出问题得到根本治理。加快腊口污水处理厂建设，抓好水阁污水处理厂改造提升。实现全市污水处理设施镇级全覆盖。开展农村生活污水治理1188个村，新增受益农户17.67万户。完善城市防洪体系，完成独流入海瓯江治理12公里、中小流域治理60公里。加快市区联城区块、城东区块供水管网建设，推进庆元、缙云、松阳、遂昌、景宁县城备用水源项目建设。加快创建节水型城市。重视水土流失防治与矿山生态治理。推进治气“五年行动”，基本淘汰黄标车，加快淘汰燃煤锅炉，完成市县城区餐饮油烟治理。加快治理丽水开发区工业废气，空气质量基本达到二类功能区标准。加快市区危废填埋场等环保配套设施建设。

　　以“四换三名”促进转型升级。加快淘汰落后产能，重点推进机械加工、医药化工等10个重点行业机器换人，加快实施100个以上重点工业技改项目。盘活存量土地3000亩，新建标准厂房10万平方米。实现电子商务零售额115亿元。推进标准强市、质量强市、品牌强市。深化“千名干部助推千家规模企业转型升级”活动，为亿元以上企业提供“一对一”服务措施，助推“个转企、小升规、规改股、股上市”，培育一批总部型、品牌型、上市型、高新型、产业联盟主导型的“五型”企业。

　　以创新驱动引领转型升级。开展国家知识产权试点城市创建。扶持创建更多的省级以上创新服务平台，推动丽水开发区、丽缙科技产业园及其他符合条件的工业园区创建高新园区，抓好丽水开发区生物医药产业孵化(加速)器、弗瑞思研究院等平台建设，新建10家以上省级企业研究院和高新技术企业研发中心。探索与中关村国家自主创新示范区战略合作，完善国家大院名校丽水产业科技服务中心运行机制，推进省级科技大市场建设试点。实施高新技术企业三年培育计划，新增高新技术企业和科技型中小企业100家以上。出台符合丽水实际的科技金融相关政策性意见。加强高层次、高技能人才队伍建设，积极引进国家和省“千人计划”、科技领军人才和项目。

　　以“五资齐抓”助推转型升级。坚持浙商、外资、央企、民资、侨资“五资齐抓”，做优、做大招商引资增量，促进产业结构调整。深度研究招商目标指向和生态优势“无缝对接”，重点研究哪些行业、产业、企业在生态优势好的地方发展更合适、更有效益，提高招商针对性。在打破周边梯度转移的被动招商模式上下功夫，积极扩大与长三角、海西区以及珠三角城市的合作。力争全市实际利用内资255亿元、外资2亿美元，浙商回归到位资金115亿元，引进大项目100个；签约山海协作产业项目100个，到位资金120亿元。

　　(四)围绕增创发展新动力，推动改革试点新突破。

　　深入推进“3+1”全国改革试点。深化农村金融改革试点，推进农村“三权”抵押贷款扩面增效，创新开展股权、水利权、宅基地使用权抵押模式，加快构建农村“六权”抵押贷款体系，同步推进产权、信用、担保服务体系的一体化。深化低丘缓坡开发利用试点，基本完成首期5.56万亩试点任务，加快配套基础设施建设，创造“地等项目、地等企业”条件。深化扶贫改革试验区建设，建立差异化搬迁补助机制，大力推进村级集体资产股份化改造和农村宅基地确权发证，大幅提高农房确权发证率。把推进国家生态文明先行示范区建设，作为具有龙头性意义的改革来抓，在“先行”和“示范”上下功夫，并统筹抓好全国水生态文明试点和庆元、景宁国家主体功能区建设试点示范。积极支持省级重点生态区县深化改革。

　　深入推进“3+1”丽水特色改革。深化社会事业综合改革，鼓励和引导社会力量举办优质社会事业，开工建设20个以上民办教育、医疗、文化、体育和养老项目，积极推进民办博物馆建设。深化生态旅游全域化综合改革，争创省级生态旅游综合改革试验区。深化丽水开发区体制机制改革，落实市直部门捆绑考核机制，促进集聚集约集群发展。推进华侨资金利用进一步破题，挖掘和释放华侨投资潜力。

　　强化改革主动性，把问题导向落到实处。尤其要以政府自身改革带动重点领域改革，积极对接上海自贸区改革和浙江省“4+6”国家战略，深化“四张清单一张网”建设，全面实行企业“零地”投资项目政府不再审批，全面实行企业独立选址项目高效审批，积极探索企业非独立选址项目市场化供给要素机制和政府不再审批办法，花更大力气把简政放权、放管结合的改革推向深入。围绕经济发展面临的突出问题推进经济综合改革，针对企业生产经营困难等突出问题，深化企业转贷应急基金、小微企业助保贷新型融资模式等改革举措，落实各项帮扶措施，更加用心用力地服务企业；针对要素配置效率问题，学习“海宁经验”，以企业绩效评价为基础，深化要素配置市场化改革；针对为发展提供可持续的资金保障问题，加大政府性资产清理和盘活力度，积极探索国资营运公司整合转型，加快探索促进社会资本进入公共设施建设领域的有效机制，推进10个以上PPP项目试点。支持丽水海关协同推进长江经济带区域通关一体化改革，支持各县(市、区)推进和争取省级以上改革试点。

　　(五)围绕推进城乡一体化，推动美丽城乡统筹协调发展。

　　坚持基础设施建设先行，打造现代综合交通枢纽。力争金丽温铁路扩能改造工程建成通车，步入“高铁时代”。加快衢宁铁路建设，力争金台铁路开工建设，推动衢丽铁路松阳至丽水段、龙浦铁路等项目前期，力争早日实现“县县通铁路”。在“县县通高速”基础上，把打造出省、出市大通道作为高速公路建设的重点。基本完成龙浦高速公路主体工程，加快金丽温高速丽水北互通项目建设，抓好龙丽温(泰)高速公路项目前期，积极谋划武松龙、遂江、庆寿等高速公路项目前期，统筹推进8个国省道和9个重要县道项目建设。推进瓯江航道整治丽水下游段工程和青田港建设，加快打造丽水出海通道。加快完成丽水机场项目前期，争取试验段和进场道路开工建设。

　　推进山水特色新型城市化。强化中心城市功能，在“一江双城”基础上，提升“北居南工中闲”内涵，持续推进“青山画城、绿水兴城、文化荣城”特色发展。加快丽水机场区块、联城区块、旧机场区块、环南明湖区块和南城开发建设，完善城市框架，增强中心城市辐射力、带动力。优化城市路网、管网体系，新建、续建城市道路8条，新开工地下空间15万平方米，新增城市园林绿地15公顷。推进市区刘祠堂背、高井弄、酱园弄等历史文化街区保护修缮工程，启动行春门城墙保护修复项目。强化市区联动共管，完善国家卫生城市精细化管理长效机制，力争获得全国文明城市提名资格。加强城市交通治堵工作。以“六个一”项目建设为重要载体，推进“一县一主题、一县一特色”差异化发展，提升美丽县城特色，基本完成第一批“六个一”项目建设。加快4个省级小城市和21个省市级中心镇组团式发展。

　　推进“美丽乡村”连线成片。坚持“点、线、面”统筹推进，整合乡村景区资源、农业产业资源和乡土文化资源，推进美丽乡村示范村和最美乡村连线成片，抓好“六边三化三美”。开展农村垃圾减量化资源化处理试点。加快打造一批体现“处州民居”特色的美丽乡村精品村，新启动建设15个示范村。加强历史文化村落保护，基本完成省级第一批历史文化村落项目，启动第二批项目。支持遂昌创建全国美丽乡村建设标准化试点县、松阳建设全国传统村落保护发展示范县。

　　(六)围绕以人为本，增进民生福祉。

　　千方百计增加城乡居民收入，促进增收富民。坚持把农民收入持续普遍较快增长作为“三农”工作的核心，大力推进产业富农、素质强农、政策惠农、改革助农“四大工程”，加快形成以家庭经营性收入为基础、工资性收入为重点、财产性收入和转移性收入比重稳步提高的农民收入快速增长长效机制。致力于“经营乡村”，大力发展“美丽经济”、“高山经济”、“林下经济”等新业态、新增长点，促进农民增收。扩大扶贫重点村资金互助会试点，加大“三农”政策性保险扶持力度。推进精准扶贫，实现精准脱贫，防止平均数掩盖大多数，基本消除家庭人均年收入低于4600元的贫困现象。全面落实促就业各项政策，实现城镇新增就业1.4万人，努力让每个有创业愿望的人都拥有自主创业的空间。逐步提高最低工资标准及城乡低保、农村五保供养补贴标准。

　　在强化社会保障中惠民。按照“保基本、兜底线、促公平、可持续”的总体要求，坚持政府新增财力三分之二以上用于民生保障。建立更加公平可持续的大社保体系，健全社会保障待遇确定机制和正常调整机制，逐步提升社会保障统筹层次。推进被征地农民基本生活保障与职工基本养老保险制度并轨，加快公办示范性养老机构建设和农村敬老院改造升级，探索医养结合、公建民营等多种模式。新建城乡社区居家养老服务照料中心240个，新增养老机构床位1770张。新开工建设保障性安居工程住房2600套，竣工2000套。积极发展社会福利、慈善和红十字事业。切实保障妇女、未成年人、老年人和残疾人合法权益。

　　在繁荣社会事业中利民。不断完善基本公共服务设施体系，完成丽水五中、市体育馆、市第二人民医院迁建等一批项目建设，加快实施市图书馆新馆、市人民医院东城院区等一批项目。全面启动教育现代化县创建，实施新一轮学前教育三年行动计划，加快省义务教育标准化学校建设，深化中小学课程改革。推动地方高校和现代职业教育发展。推进卫生强县创建，加快人口健康信息化建设，深化县级公立医院改革，启动龙泉、松阳、云和、景宁分级诊疗试点。广泛开展群众性精神文明创建，完善“文化订制”新模式，深化乡村春晚、文化四季、乡村文化艺术节等文化惠民品牌活动。支持景宁打造全国畲族文化总部，促进民族区域发展。广泛开展全民健身活动，积极承办一批国际国内赛事。统筹抓好民族宗教、人口计生、广播电视、新闻出版、方志、档案、统计、气象等方面工作，促进各项社会事业协调发展。

　　在创新社会治理中安民。强化社会主义核心价值观及社会公德、社会诚信体系建设。深入实施夺“金鼎”三年平安计划。完善社会矛盾大调解体系，有效化解企业改制、征地拆迁、医疗等领域的矛盾纠纷，切实解决群众合理信访诉求。鼓励和引导社会组织参与社会事务管理。落实户籍制度改革措施，改进流动人口服务管理。建立农村留守老人、儿童、妇女的关爱服务体系。强化安全生产责任倒查、基层基础和行政执法，完善食品药品、消防安全责任体系，健全防灾减灾体系。健全社会应急联动机制。深化“天网工程”，强化立体化社会治安防控。着力防范和化解企业资金链、担保链风险，着力化解银行不良贷款。切实做好国家安全、国防建设、人民防空和双拥优抚工作。

　　在办好实事工程中为民。坚持尽力而为、量力而行，明确任务表、时间表、责任表，努力办好全市十方面大事和市区十方面实事。全市十方面大事为：以“五水共治”为突破口强化生态环境治理，深入推进“三改一拆”，深化“六边三化三美”，抓好生态精品农业特色发展，抓好生态制造业创新发展，抓好生态旅游业全域化发展，大力发展信息经济，打造现代综合交通枢纽，抓好要素保障，全面推进农村产权制度改革。市区十方面实事为：民生水务、城市畅通、小区改造、教育保障、医疗服务、文化惠民、就业帮扶、助老扶残、食品安全、便民服务。

三、加快建设法治政府，提升政府治理能力

　　深入学习贯彻《中共中央关于全面推进依法治国若干重大问题的决定》及省委、市委的部署要求，加快建设职能科学、权责法定、执法严明、公开公正、廉洁高效、守法诚信的法治政府。

　　坚持依法行政，强化政府执行力。推进机构、职能、权限、程序、责任法定化，坚持法定职责必须为、法无授权不可为，勇于负责、敢于担当，着力解决行政不作为、慢作为和乱作为问题。深化行政执法体制改革，整合执法主体，推进综合执法，着力解决权责交叉、多头执法等问题。全面落实行政执法责任制，健全行政执法裁量权基准制度、公示制度和重大行政行为报备制度。完善行政裁决执行制度。对发展中的疑难复杂问题，继续推进攻坚。

　　坚持公开行政，强化政府公信力。自觉接受人大、政协和社会各界监督，主动听取民主党派、工商联、无党派人士和人民团体意见建议。认真做好人大代表建议、批评和意见及政协提案办理工作。落实好公众参与、专家论证、风险评估、合法性审查、集体讨论决定的重大行政决策法定程序。建立行政机关内部重大决策合法性审查机制，普遍建立政府法律顾问制度。坚持问政于民、问计于民、问需于民，坚持以公开为常态、不公开为例外原则，推进决策公开、执行公开、管理公开、服务公开、结果公开。

　　坚持科学行政，强化政府运筹力。推进“智慧政务”试点，建设大数据中心，着力构建“平台+数据”的政务云模式，加快“企业运行数据系统”、“自然人数据系统”等一批应用项目开发和推广。创新政府管理服务方式，推动公共资源市场化配置，推进政府向社会力量购买服务。抓好部门职能和部门内部职能的归并整合，构建大联办、大代办、大中介“三大服务品牌”，全力打造名副其实的“最佳投资环境之市”。

　　坚持从严治政，切实提高拒腐防变能力。巩固和拓展党的群众路线教育实践活动成果，持之以恒落实中央八项规定和省委“28条办法”、“六个严禁”以及市委实施意见，落实省委、市委“十大整改行动”，加大正风肃纪力度，在抓常、抓细、抓长上下功夫，切实做到“三严三实”。推进行政问责，强化政府工作目标管理责任制和考核激励机制，确保重点工作、重大项目落到实处。加强行政权力监督制约，加强审计监督和行政监察，完善廉政风险防控机制，严防领导干部利益冲突和利益输送问题。坚持查处大要案和查处发生在群众身边的腐败问题并举。健全厉行节约反对浪费制度体系，完成公务用车制度改革，严控“三公”经费支出，把有限的财力用到最有需求、最有效率的地方，特别是群众最迫切需要解决的问题上。

　　各位代表，丽水的发展正处在关键时期，挑战虽多，路在脚下。让我们在省委、省政府及市委的坚强领导下，坚定“丽水自信”，凝心聚力，攻坚克难，开拓进取，为开创“绿水青山就是金山银山”的发展新境界，加快建设美丽幸福新丽水而努力奋斗！
